# Supplementary material for: Curated and harmonised transcriptomics datasets of interstitial lung diseases
Source: Data Brief. 2025 Oct 14;63:112139. doi: 10.1016/j.dib.2025.112139 (PMC12581653; doi:10.1016/j.dib.2025.112139)
Supplement: Supplementary file 1 [file mmc1.zip › Supplementary_material/RNA-seq/fastQC reports/GSE169500/SRR14057112_fastqc.html]

SRR14057112.fastq.gz FastQC Report 

FastQC Report

Mon 5 Feb 2024  
SRR14057112.fastq.gz

## Summary

- Basic Statistics
- Per base sequence quality
- Per sequence quality scores
- Per base sequence content
- Per sequence GC content
- Per base N content
- Sequence Length Distribution
- Sequence Duplication Levels
- Overrepresented sequences
- Adapter Content

## Basic Statistics

| Measure | Value |
| --- | --- |
| Filename | SRR14057112.fastq.gz |
| File type | Conventional base calls |
| Encoding | Sanger / Illumina 1.9 |
| Total Sequences | 22608232 |
| Sequences flagged as poor quality | 0 |
| Sequence length | 25-377 |
| %GC | 50 |

## Per base sequence quality

## Per sequence quality scores

## Per base sequence content

## Per sequence GC content

## Per base N content

## Sequence Length Distribution

## Sequence Duplication Levels

## Overrepresented sequences

| Sequence | Count | Percentage | Possible Source |
| --- | --- | --- | --- |
| ACCAATGTCATAGGGTGCAATATCTACAATAGGTAGTCTCACAGCCTTGC | 331595 | 1.4667002709455563 | No Hit |
| ACACTGAGGACTCTGTTCCTCCCCTTTCCGCCTAGGGGAAAGTCCCCGGA | 273171 | 1.2082811252113832 | No Hit |
| GGGAATGTCTACGTGCGTATGCACGTGGCACTCTCTGCCCGAGGTCCGGG | 224263 | 0.9919528426636811 | No Hit |
| TGGTTGCACGAAACACACTGGGGAATGGAGCAAAACAGTCTTTGAATATC | 210202 | 0.9297586825895984 | No Hit |
| TTCTTGTAGATGGCTGCTCTAAAAAGACAAATGAATGGGGAAAGACAATC | 172424 | 0.7626602557864763 | No Hit |
| AGAGCATGACCGATGGATTCCAGTTCGAGTATGGCGGCCAGGGCTCCGAC | 159747 | 0.7065877597151339 | No Hit |
| CAATATCAAGGAAGGGCAGGCGTGATGGCTTATTTGTTTTGTATTCAATG | 159239 | 0.7043407905580586 | No Hit |
| GGGCCACGAGCTGAGTGCGTCCTGTCACTCCACTCCCATGTCCCTTGGGA | 131056 | 0.5796826571843389 | No Hit |
| AAGCTCAGGGAGAGCCCTGTTAGGGCCGCCTCTGGCCCTAGTCTCAGACC | 121180 | 0.5359994536503341 | No Hit |
| ATGTTCTGGGAGGCCTCGGTGGACATCAGGCGCAGGAAGGTCAGCTGGAT | 116168 | 0.5138305374785609 | No Hit |
| GCTGCTACAGGAGAATAGCAGACAGGTATAGTTAAGAAACAACTTTATTT | 91133 | 0.40309653581049587 | No Hit |
| CTAAGTGCTGTGTTGTCGTTCCCCCTGCTTAAAATAAAGTTGTTTCTTAA | 78833 | 0.34869157393643163 | No Hit |
| TCTGCAATGCAATCACAATGCCCAAACTAGACCTGCCATTTCTCACACAC | 72343 | 0.31998521600450663 | No Hit |
| TGTTGTGCTAAACGCTGGGAGGGTACAAGTTGGTCATTCCTAAATCTGTG | 61842 | 0.2735375327004783 | No Hit |
| TGGCTCTCCCAGAGGCAGAAGGACAATGGCTGTTTCAGGAGCTCTGGGTC | 61217 | 0.2707730529304547 | No Hit |
| TCTGTCTTCTTCAGTTTCGACTTATCGAATTTCTCGATCTCAGCCATATC | 56558 | 0.2501655149327908 | No Hit |
| GAGAGGGTCACTTCATCTTCTACTCCTCCCTTTATGGCATTGTTGAGCAG | 52115 | 0.23051338114364714 | No Hit |
| CAGAAGCTCTTTATGTCAAACTTCTCAAGTTGATTGACCTCCAGGTAATA | 50205 | 0.22206513096645505 | No Hit |
| ACACTGAGGACTCTGTTCCTCCCCTTTCCGCCTAGGGAAAGTCCCCGGAC | 50102 | 0.22160954470035518 | No Hit |
| ACACCCACCGCAACTGTCTGTCTCATATCACGAACAGCAAAGCGACCCAA | 50053 | 0.22139280948638534 | No Hit |
| TTCCCCCTGCGCATGCGGGACTGGCTCAAGAACGTCCTGGTCACCCTGTA | 49546 | 0.21915026349694217 | No Hit |
| TGGTTCCTGGCAAGCCCATGTGTGTTGAGAGCTTCTCAGACTATCCACCT | 48588 | 0.21491286890545003 | No Hit |
| AGCGGTTCCGCTGCCCTGAGGCACTCTTCCAGCCTTCCTTCCTGGGCATG | 46757 | 0.2068140489711889 | No Hit |
| TTTACACCTGCTAGGGTGTTCAAAGGTCAGTGCTATAGAAATTCAGTATC | 46643 | 0.2063098078611366 | No Hit |
| ACCCGCAGCTTCTGCTTCTCAGTCAGAAGGTTGTTGTCCTCATCCCTCTC | 46050 | 0.2036868694553382 | No Hit |
| TCACACTTCATGATGGAGTTGAAGGTAGTTTCGTGGATGCCACAGGACTC | 45947 | 0.20323128318923833 | No Hit |
| TGTTCCCTCTCCTCATGAGATTGGTGAAGAAAGTATTTGGCAAAGTTCTT | 43550 | 0.19262895037524386 | No Hit |
| TACTCGTGCGCCTCGCTTCGCTTTTCCTCCGCAACCATGTCTGACAAACC | 42550 | 0.1882057827432061 | No Hit |
| TGTCCATGTCTTACTACTTTGACCGCGATGATGTGGCTTTGAAGAACTTT | 40250 | 0.17803249718951927 | No Hit |
| ATGTTCTGGGAGCCTCGGTGGACATCAGGCGCAGGAAGGTCAGCTGGATG | 39019 | 0.17258757783448084 | No Hit |
| TGTCCTATAACAAGCTTAAAAACATACCAACTGTCAATGAAAACCTTGAA | 37269 | 0.16484703447841476 | No Hit |
| TGCGAAAAGTTACTTTCAATGGACTGAACCAGATGATTGTCATAGAACTG | 36973 | 0.16353777685933157 | No Hit |
| TGTGCCCTGGAAGCGGAATGGAAACTTGCAGGGCTGTCCTTCAGCGTTGC | 34695 | 0.15346180099354959 | No Hit |
| AATACCAAGACCTGCTCAATGTTAAGATGGCCCTTGACATTGAGATTGCC | 34140 | 0.15100694295776865 | No Hit |
| TCTTTCTGGCCTGGAGGCTATCCAGCGTACTCCAAAGATTCAGGTTTACT | 31242 | 0.13818860316012327 | No Hit |
| CAGTAGCACCAACGTTGATGAGGTCGTGAGTCAGGGGTCCTAGGAAGCTC | 31199 | 0.13799840695194565 | No Hit |
| ATCGCCTGCGACAAGCCACAGCAGGTGCGCAACATGACGCTGTTCTCCGA | 30030 | 0.13282772399009352 | No Hit |
| AAAGCCAAGAAAACCAACGATGCCAGATACTGAATTTCTATAGCACTGAC | 29684 | 0.13129730798940847 | No Hit |
| GAAAAGTTTGGAAGAGGCAGAGAAATCCTGCTCTCCTCGCCTTCCAGCAG | 28591 | 0.1264627857675912 | No Hit |
| GATGGCAAGTACGGCTTCTGTCCCCATGAAGCCCTGTTCACCATGGGCGG | 25998 | 0.11499351209771733 | No Hit |
| TGGAAAGCCCCATTTCAATTCCTGAGCTCTTCAGCGGATTGGTGCCCAGT | 24835 | 0.10984936814165742 | No Hit |
| ATTCTTCCTACCCATGAGCATGGAATGTTCTTCCATTTGTTTGTATCCTC | 24551 | 0.1085931885341587 | No Hit |
| TCCCTTTCTCCATGCATGAATTTTCAATGCAGACAGCTGTACGATATACG | 23956 | 0.10596140379309626 | No Hit |
| ATTTTCCCAGGTGGGGCCTGCCTGGGGAAAGCTTGTGGCCGGAAGAGAAA | 23257 | 0.10286960961830187 | No Hit |
| GGCAGAGGACGTCCTCCATGTCATCGATGAACTTCTCAGCGACCAGGTCG | 23027 | 0.10185228106293319 | No Hit |
| GGTTATAATCATTGGCAATGTTGTAATCAACAACCCATGTTATTTCTACA | 22874 | 0.1011755364152314 | No Hit |
| TCTTCCAGCCTACTTGTGTCTTCCAACAAAGGTACGTCTTCCAACAAAGG | 22609 | 0.1000033969927414 | No Hit |

## Adapter Content

Produced by FastQC (version 0.11.7)
